# Molecular phylogeny and morphology reveal three new species and two new records of *Trichoderma* (*Hypocreaceae*, *Hypocreales*) from China

**DOI:** 10.3897/mycokeys.139.198944

**Published:** 2026-08-21

**Authors:** Fang-Wei Lou, Bao-Zheng Chen, Li-Man Tan, Jia-Chen Xiao, He-Fa Liao, Jian-Hua Hou, Zhu-Liang Yang, Guo-Dong Li, Yuan-Bing Wang

**Affiliations:** 1 School of Chinese Materia Medica, Yunnan University of Traditional Chinese Medicine, Kunming 650500, China State Key Laboratory of Phytochemistry and Natural Medicines, Kunming Institute of Botany, Chinese Academy of Sciences Kunming China; 2 State Key Laboratory of Phytochemistry and Natural Medicines, Kunming Institute of Botany, Chinese Academy of Sciences, Kunming 650201, China Yunnan Key Laboratory for Fungal Diversity and Green Development, Kunming Institute of Botany, Chinese Academy of Sciences Kunming China; 3 Yunnan Key Laboratory for Fungal Diversity and Green Development, Kunming Institute of Botany, Chinese Academy of Sciences, Kunming 650201, China School of Chinese Materia Medica, Yunnan University of Traditional Chinese Medicine Kunming China; 4 Qinghai Agricultural Products Quality and Safety Testing Center, Xining 810001, China Qinghai Agricultural Products Quality and Safety Testing Center Xining China

**Keywords:** Morphological characteristics, novel species, *Ophiocordyceps sinensis*, phylogenetic analyses, taxonomy

## Abstract

Species of *Trichoderma* are globally distributed across diverse ecosystems. In this study, four *Trichoderma* strains were isolated from *Ophiocordyceps jinguangensis*, two from *O. sinensis*, and two from *Dracaena* sp. To determine their taxonomic placement, maximum likelihood and Bayesian inference analyses of ITS, *tef1-α*, and *rpb2* sequences, along with morphological examinations, were conducted. Integrating phylogenetic and morphological evidence, these strains were identified as three new species, *Trichoderma diqingense*, *T. dracaenae*, and *T. yuxiense*, which are distributed in three distinct clades of *Trichoderma* (i.e., *Harzianum*, *Viride*, and *Polysporum*, respectively), and two known species, *T. fertile* and *T. paraviridescens*, which are newly recorded from the substrate *O. sinensis*. Descriptions and illustrations of all species are provided. These findings expand the understanding of *Trichoderma* species and substrate diversity, providing a taxonomic basis for future studies.

## Introduction

The genus *Trichoderma* belongs to *Hypocreaceae*, *Hypocreales*, *Sordariomycetes*, and *Ascomycota* (https://www.indexfungorum.org/). The genus has undergone significant revision since it was established by Persoon in 1794 (Persoon 1794). The sexual stages had long been placed in *Hypocrea*; because of their pronounced morphological differences from the asexual stages, they were typically treated as separate taxa in early studies (Tulasne and Tulasne 1865; Chaverri and Samuels 2003; Samuels 2006). This dual nomenclature persisted until the adoption of the “One Fungus = One Name” principle, whereby *Hypocrea* was suppressed in favor of *Trichoderma* on the basis of nomenclatural priority and the considerable practical importance of the latter (Rossman et al. 2013; Turland et al. 2018). Subsequently, 46 species formerly placed in *Hypocrea* were transferred to *Trichoderma* by Jaklitsch and Voglmayr (2013). Since then, *Trichoderma* has been widely accepted as the unique generic name in taxonomy, phylogenetics, and applied research, particularly in the biocontrol of fungal diseases and as a source of industrial enzymes (Rossman et al. 2013). To date, more than 500 species have been formally described in the genus (Zhao et al. 2025), and these are now classified into 17 phylogenetic lineages viz., *Harzianum*, *Helicum*, *Chlorosporum*, *Spinulosum*, *Ceramicum*, *Strictipile*, *Hunanense*, *Spirale*, *Stromaticum*, *Longibrachiatum*, *Semiorbis*, *Viride*, *Polysporum*, *Brevicompactum*, *Deliquescens*, *Hypocreanum*, and *Psychrophilum* (Jaklitsch and Voglmayr 2015; Chen and Zhuang 2017; Bustamante et al. 2021; Sales et al. 2025).

Species of *Trichoderma* are found worldwide across diverse ecosystems, from grasslands and forests to wetlands, saline-alkaline soils, and deserts, and have been isolated from a variety of substrates, including decaying wood, leaves, bark, roots, and mushrooms (Samuels et al. 2006; Hatvani et al. 2007; Qiao et al. 2018; Bai et al. 2023). By suppressing plant pathogens via mycoparasitism, nutrient competition, and the induction of plant immunity, they have become some of the most widely used biological control agents in agriculture (Guzmán-Guzmán et al. 2017; Li et al. 2020; Dou et al. 2022; Xie et al. 2023; Guzmán-Guzmán et al. 2025). Among registered fungal biocontrol agents, *Trichoderma* accounts for approximately 60% of the total, with more than 50 *Trichoderma*-based formulations successfully developed worldwide (Woo et al. 2006; Lorito et al. 2010; Saravanakumar and Kathiresan 2014; Bischof et al. 2016; Saldaña-Mendoza et al. 2023; Ye et al. 2023). As the largest phylogenetic group within the genus, the *Harzianum* clade comprises approximately 95 species. Many of these species have notable biocontrol functions, e.g., *T. harzianum*, *T. afroharzianum*, *T. endophyticum*, and *T. azevedoi* (Chaverri et al. 2015; Barrera et al. 2021; Cao et al. 2022; Ismaiel et al. 2024; da Veiga et al. 2026). Numerous *Trichoderma* species have been reported in close association with plants or other microorganisms, playing significant roles in promoting plant growth, modulating plant defense responses, enhancing photosynthetic efficiency, and improving crop tolerance to abiotic stresses (Harman et al. 2019; Ferreira and Musumeci 2021; Guzmán-Guzmán et al. 2025; Santos and Santos 2025). In addition, many species exhibit strong cellulolytic activity and are widely used in diverse industrial sectors, including bioenergy, textile processing, papermaking, detergents, and feed production (Mandels and Reese 1957; Harkki et al. 1989; Mandels and Eveleigh 2009; Fischer et al. 2021). Recently, *Trichoderma* has also attracted considerable attention for its potential applications in pollution remediation, nanomaterial synthesis, and the development of functional fermentation products (Saleh et al. 2018; Abdel-Aty et al. 2019; Ye et al. 2023).

In this study, six *Trichoderma* strains were isolated from *Ophiocordyceps* spp. and two strains were isolated from *Dracaena* roots, all collected from China. Multilocus phylogenetic analyses were performed using the internal transcribed spacer region (ITS), the translation elongation factor 1-alpha gene (*tef1-α*), and the RNA polymerase II second-largest subunit gene (*rpb2*), combined with morphological observations of colonies, conidiophores, phialides, conidia, and chlamydospores. Detailed descriptions, illustrations, and taxonomic notes are provided for these species.

## Materials and methods

### Sample collection and strain isolation

Twenty samples, including *Ophiocordyceps* specimens and fresh *Dracaena* roots, were collected during 2024–2025 from Yunnan Province, Qinghai Province, Xizang Autonomous Region, and Guangxi Zhuang Autonomous Region, China. For each sample, detailed sampling information was recorded, including the collection date, locality, geographic coordinates, and altitude. Samples were individually placed in sterile polyethylene zip-lock bags, maintained in a cooled container with ice packs, and transported to the laboratory for further processing.

Fungal strains from the roots of *Dracaena* sp. were isolated using a previously described method (Fan et al. 2025). Prepared tissue segments were placed on plates containing potato dextrose agar medium (PDA; prepared in-house: potato, 200 g/L; dextrose, 20 g/L; and agar, 18 g/L) supplemented with penicillin and streptomycin (each at 100 mg/L; Sangon Biotech, Shanghai, China). Hyphal tips emerging from the tissue segments were then transferred to fresh PDA plates for purification. The purification procedure was repeated for two to three successive transfers to eliminate potential contaminants.

Samples of *Ophiocordyceps* were processed for isolation using a modified grinding method following Jaklitsch et al. (2005). Samples were rinsed with sterile water to remove debris, sterilized in 75% ethanol for 30–60 s, and treated with 30% H_2_O_2_ for 2–5 min until bubbling ceased or only a few evenly dispersed bubbles remained. After three washes with sterile water (1 min each), they were blotted dry on sterile filter paper. Tissue segments were then excised with sterilized blades. Different tissues were separated with a flame-sterilized blade and ground at two speeds to yield coarse and fine fractions. The coarse fraction was plated directly onto PDA. The fine fraction was serially diluted (1×–5×) according to Zhang et al. (2010). An aliquot of the tissue suspension was suspended in ca. 10 μL of sterile water to obtain a 1× homogenate and then serially diluted to the target concentrations. Aliquots (50–100 μL) of each dilution were spread onto PDA plates in triplicate for each sample. The inoculated plates were incubated at 25 °C in a constant-temperature incubator (HWS-250B; Hefei Youke Instrument Equipment Co., Ltd., Hefei, China), and purification was performed following the same procedure described above. Pure cultures were examined for macromorphological characteristics, and isolates with rapid growth, sparse or floccose aerial mycelium, and green or white conidia were tentatively identified as *Trichoderma* (Bissett 1991a). Representative isolates were subcultured for subsequent experiments. All *Trichoderma* strains isolated in this study were deposited in the Fungal Culture Collection of the Kunming Institute of Botany, Chinese Academy of Sciences, China (KUNCC). Dried cultures were deposited in the Cryptogamic Herbarium of the Kunming Institute of Botany, Chinese Academy of Sciences, China (HKAS).

### Morphological observations

Colony characters were recorded on PDA, synthetic nutrient-poor agar (SNA; prepared in-house: KH_2_PO_4_, 1.0 g/L; KNO_3_, 1.0 g/L; MgSO_4_·7H_2_O, 0.5 g/L; KCl, 0.5 g/L; glucose, 0.2 g/L; agar, 20 g/L; pH 7.0) (Fan et al. 2025), and oatmeal agar (OA; Shanghai Ruichu Biotechnology Co., Ltd., Shanghai, China). Strains preserved at 4 °C were reactivated on PDA at 25 °C in the same incubator. Once the colonies had covered the plates after 2–3 days, agar plugs were excised from the colony margins using a sterile 5 mm cork borer. The plugs were inoculated near the margins of PDA, SNA, and OA plates and incubated at 20 °C. Colony characters on each medium, including colony diameter, color, texture, diffusing pigment production, and odor, were examined and recorded after 7 days.

For microscopic observations, mycelial plugs (ca. 5 × 5 mm) were excised from reactivated cultures with a sterile inoculating spatula and transferred to SNA plates. Cultures were incubated at 25 °C in the dark for 3–5 days to induce sporulation (i.e., conidiation). For microscopic examination, a drop of sterile water was placed on a clean microscope slide, and a small number of hyphae and conidiophores were transferred into the droplet with a sterile dissecting needle, gently dispersed, and covered with a coverslip. Morphological characteristics were examined using an Olympus BX53 microscope (Olympus Corporation, Tokyo, Japan) with differential interference contrast (DIC) optics. For each strain, at least 30 conidiophores, phialides, and conidia were randomly selected and measured for length (l) and width (w), and the *l/w* ratio was calculated. Measurements are presented as (*a*–) *b*–*c* (–*d*), where “*a*” and “*d*” represent the minimum and maximum values, respectively, and “*b*–*c*” represents the range encompassing at least 95% of the measurements. “*n*” indicates the total number of measurements (Zhang et al. 2022).

### DNA extraction and PCR amplification

Genomic DNA was extracted from fresh mycelium grown for 5–7 days using the Ezup Column Fungal Genomic DNA Extraction Kit (Sangon Biotech, Shanghai, China) according to the manufacturer’s instructions. Polymerase chain reaction (PCR) amplifications were performed in a T Series Multi-Block Thermal Cycler (LongGene Scientific Instrument Co., Ltd., Hangzhou, China) using the following primer pairs: EF1-728F (CATCGAGAAGTTCGAGAAGG) and TEF1-LLErev (AACTTGCAGGCAATGTGG) for *tef1-α* (Jaklitsch and Voglmayr 2015), RPB2-5F (GAYGAYMGWGATCAYTTYGG) and RPB2-7cr (CCCATRGCTTGYTTRCCCAT) for *rpb2* (Liu et al. 1999), and ITS5 (GGAAGTAAAAGTCGTAACAAGG) and ITS4 (TCCTCCGCTTATTGATATGC) for the ITS region (White et al. 1989). PCR cycling conditions and reaction mixtures were based on the descriptions provided by Wang et al. (2025). Amplification products were sent to Beijing Qingke Biotechnology Co., Ltd. (Beijing, China) for Sanger sequencing.

### Phylogenetic analyses

Phylogenetic analyses were performed using ITS, *tef1-α*, and *rpb2*, which are the core loci commonly used for species identification and phylogenetic inference in *Trichoderma* (Cai and Druzhinina 2021). In this study, the quality of the newly generated sequences was first evaluated using MEGA7 (version 7.0), and the sequences were then assembled into consensus sequences with SeqMan (Lasergene v14.1; DNASTAR, Madison, WI, USA). For preliminary identification, BLASTn searches were performed against the NCBI GenBank database to retrieve closely related sequences, and representative sequences of this genus were systematically collected to construct an initial phylogenetic tree. Furthermore, a subset of sequences, particularly those closely related to the strains obtained in this study, was selected to serve as a representative framework. Reference sequences were retrieved from GenBank, and *Hypomyces chiangraiensis*MFLUCC 24-0216 was selected as the outgroup. GenBank accession numbers for all ITS, *tef1-α*, and *rpb2* sequences used in this study are provided in Suppl. material 1: tables S1, S2. For each locus, sequences were initially aligned using MAFFT v7 with the “--auto” parameter (Rozewicki et al. 2019). The resulting alignments were then checked and adjusted manually in MEGA7 (Kumar et al. 2016), with ambiguously aligned regions, poorly aligned positions, and terminal regions of uncertain alignment trimmed. The edited alignments were concatenated into a combined dataset for phylogenetic analyses.

Phylogenetic trees were constructed using maximum likelihood (ML) and Bayesian inference (BI) methods. ModelFinder (Kalyaanamoorthy et al. 2017) was used to identify the best-fitting nucleotide substitution models for the ML and BI analyses under the Akaike Information Criterion (AIC) and Bayesian Information Criterion (BIC), respectively. The number of nucleotide positions and the best-fitting substitution models for the ML and BI analyses are summarized in Suppl. material 1: table S3. The ML analysis was conducted in IQ-TREE v2.1.3 (Nguyen et al. 2015) under a partitioned model (Chernomor et al. 2016), and branch support was evaluated with 1,000 ultrafast bootstrap replicates (Hoang et al. 2018). The BI analysis was performed in MrBayes v3.2 (Ronquist et al. 2012), with four Markov chains run for 20 million generations. The first 25% of sampled trees were discarded as burn-in, and a majority-rule consensus tree was generated. The final phylogenetic tree was visualized and edited using FigTree v1.4.4 and ChiPlot (https://chiplot.online/).

## Results

### Phylogenetic analyses

**Figure 1.**
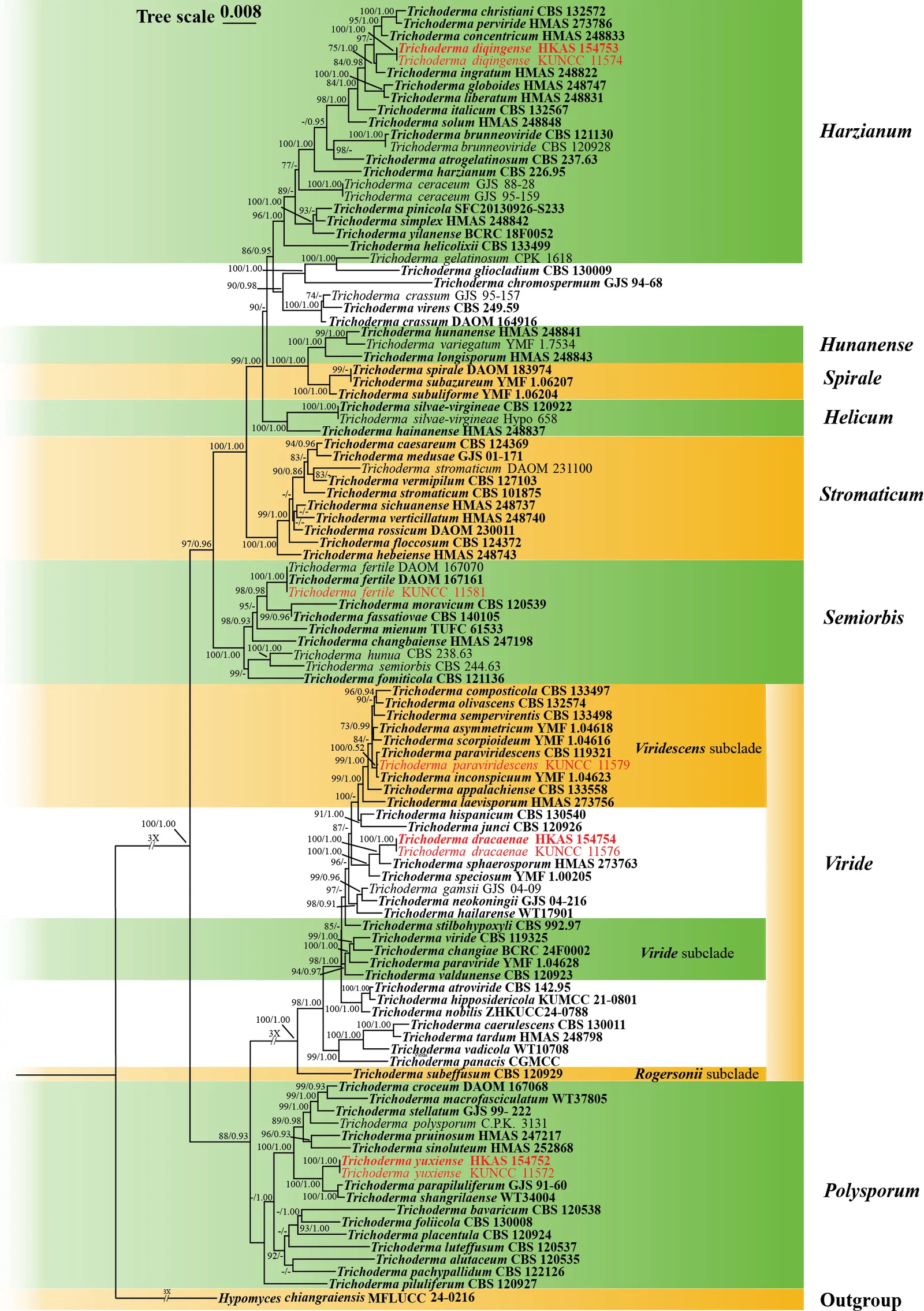
Phylogenetic tree of *Trichoderma* inferred from the combined ITS, *tef1-α*, and *rpb2* dataset. Newly isolated strains are highlighted in red. Maximum likelihood bootstrap values (ML-BS ≥ 70%) and Bayesian posterior probabilities (BI-PP ≥ 0.90) are indicated at the nodes as ML-BS/BI-PP. Bold denotes the type strains/species.

In this study, a total of eight *Trichoderma* strains were isolated from *Ophiocordyceps jinguangensis*, *O. sinensis*, and *Dracaena* sp. For the initial phylogenetic tree, sequences from three loci for 541 representative strains covering 473 species were collected and analyzed, resulting in a concatenated supermatrix of 2,594 characters (ITS: 559; *tef1-α*: 982; *rpb2*: 1,053), from which an ML phylogenetic tree was constructed (Suppl. material 1: table S1; Suppl. material 2). The phylogenetic analysis revealed that six of the eight strains were potential new species, while the remaining two were tentatively assigned to known species. In addition, these results were further verified by sequence identity assessments based on the criterion of “*rpb2*_99_ ≈ *tef1-α*_97_” proposed by Cai and Druzhinina (2021). To better present the phylogeny of the strains obtained in this study, sequences from three loci for 90 species represented by 96 strains were selected and analyzed. As a result, a concatenated matrix of 2,437 characters was obtained, including 385 from ITS, 994 from *tef1-α*, and 1,058 from *rpb2*. Phylogenetic analyses showed that the topologies inferred from ML and BI were congruent (Fig. 1; Suppl. material 1: table S2). The eight strains were assigned to four clades of *Trichoderma*, namely the *Harzianum*, *Semiorbis*, *Viride*, and *Polysporum* clades. Within the *Harzianum* clade, strains KUNCC 11573 and KUNCC 11574 formed a distinct monophyletic lineage closely related to *T. christiani*, *T. concentricum*, *T. ingratum*, and *T. perviride*. Strain KUNCC 11581, placed within the *Semiorbis* clade, grouped with the reference strains CBS 339.93 and DAOM 167070 and was identified as *T. fertile*. Strain KUNCC 11579, placed within the *Viride* clade, grouped with the reference strain CBS 119321 and was identified as *T. paraviridescens*. Strains KUNCC 11575 and KUNCC 11576 formed a distinct monophyletic lineage within the *Viride* clade and were resolved as closely related to *T. speciosum* and *T. sphaerosporum*. Within the *Polysporum* clade, strains KUNCC 11571 and KUNCC 11572 formed a monophyletic lineage closely related to *T. parapiluliferum* and *T. shangrilaense*.

### Taxonomy

#### Trichoderma diqingense

Taxon classificationFungiHypocrealesHypocreaceae

F. W. Lou, Zhu L. Yang & Y. B. Wang sp. nov.

57D656F3-AD7C-51A4-BE09-C80C5846DF5E

Fungal Names: FN 573937

Fig. 2

##### Etymology.

Named after the location, Diqing Tibetan Autonomous Prefecture, where this species was collected.

**Figure 2.**
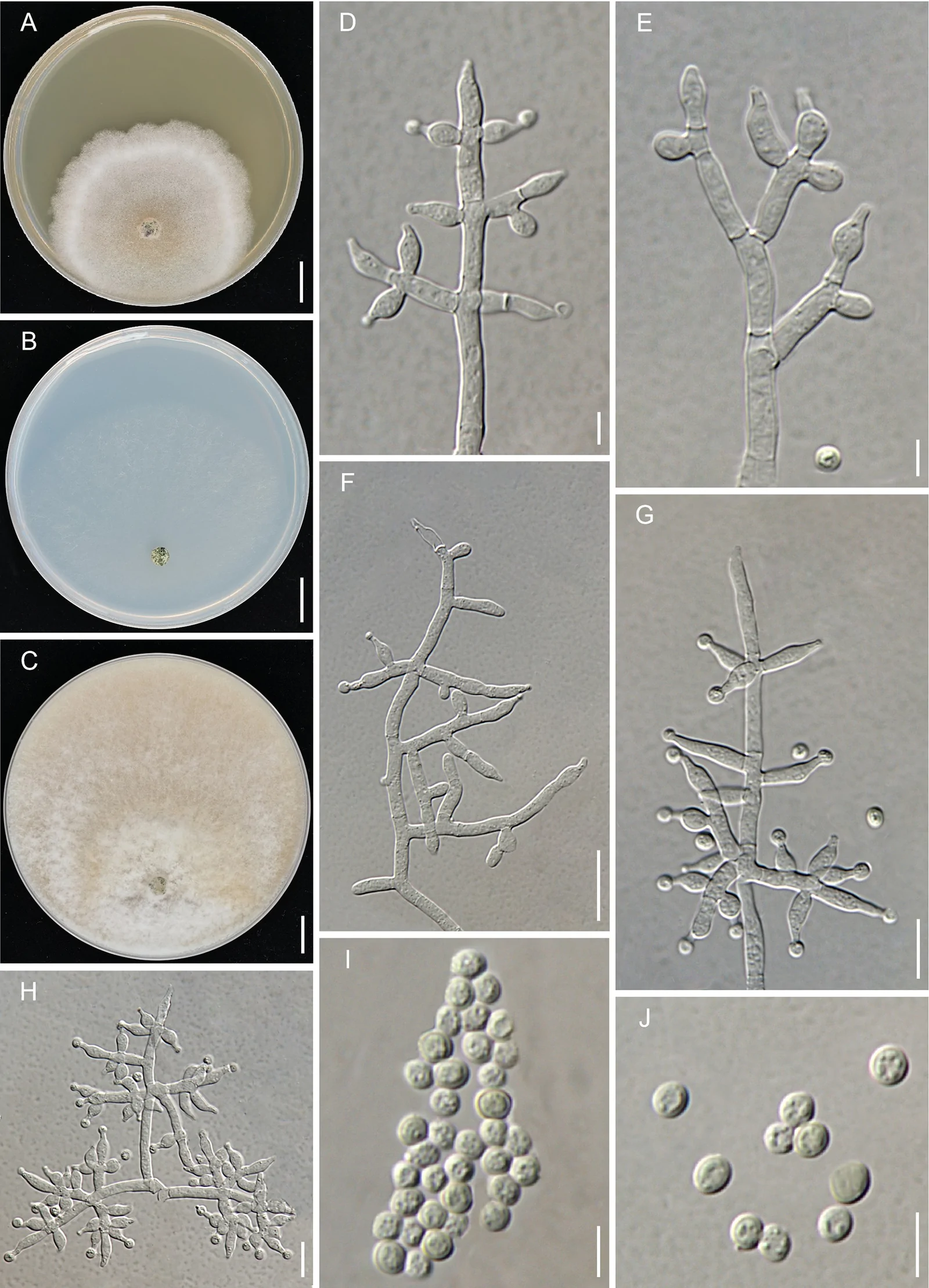
*Trichoderma diqingense* (KUNCC 11573, ex-type living culture). **A–C**. Cultures on different media after 7 d at 20 °C (A, PDA; B, SNA; C, OA); **D–H**. Conidiophores and phialides; **I, J**. Conidia. Scale bars: 1 cm **(A, B)**; 2 cm **(C)**; 10 μm **(D, E, I, J)**; 30 μm **(F)**; 20 μm **(G, H)**.

##### Type.

China • Yunnan Province, Diqing Tibetan Autonomous Prefecture, Shangri-La City, 27°33'N, 99°49'48"E, altitude 3220 m, isolated from the sclerotium of *O. sinensis*, 16 July 2025, Fang-Wei Lou (holotype HKAS 154753; ex-type living culture KUNCC 11573).

##### Diagnosis.

*Trichoderma diqingense* is similar to *T. concentricum*, *T. ingratum*, *T. perviride*, and *T. christiani* but differs by having pyramidal conidiophore branching, narrower lageniform phialides, slightly rough-walled ellipsoidal to globose conidia, by lacking chlamydospores and a coconut-like odor.

##### Description.

**Sexual morph**: Unknown. **Asexual morph: *Conidiophores*** with branches arranged in a pyramidal structure; primary branches mostly in whorls of three, occasionally opposite or solitary, each branch terminating in a whorl of 2–5 phialides or bearing solitary phialides. ***Phialides*** lageniform, usually arising in whorls of 1–5 on the main axis and branches, (5.3–)6.2–12.4(–17.3) × (2.1–)2.2–3.2(–3.5) μm (av. 8.6 × 2.7 μm), *l/w* (2.0–)2.1–4.9(–5.9) (av. 3.2). ***Conidia*** slightly rough-walled, ellipsoidal to globose, light green, (2.0–)2.4–3.5(–4.2) × (1.4–)1.5–2.9(–3.1) μm (av. 2.9 × 2.2 μm), *l/w* (1.1–)1.1–1.8(–1.8) (av. 1.3). ***Chlamydospores*** not observed.

##### Cultural characteristics.

Colony radius on PDA reached 5.3–5.6 cm after 7 d at 20 °C, covering the plate after 14–15 d. Colonies well defined, cream-white; colony margin irregular, slightly undulate, distinct; mycelia dense, abundant, forming a distinct zonate band along the colony margin. Conidiation commenced after 15 d, producing light-green conidia in aggregated minute pustules arranged in two distinct concentric rings. No diffusing pigment or distinct odor. Colony radius on SNA reached 4.4–4.6 cm after 7 d at 20 °C, covering the plate after 15–16 d. Colonies white, thin, flat; colony margin regular, distinct; mycelia sparse, pale white. Conidiation commenced after 10 d as minute white pustules; conidia gradually became light green after 12 d, with pustules developing along the colony margin, globose to irregular in shape. No diffusing pigment or distinct odor. Colony radius on OA reached 5.9–6.2 cm after 7 d at 20 °C, covering the plate after 6–7 d. Colonies white, with indistinct margins; mycelia abundant, floccose, with loose hyphae. Colony morphology similar to that on PDA. Conidiation commenced after 16 d as light-green, cottony pustular aggregations, without obvious concentric rings. No diffusing pigment or distinct odor.

##### Distribution.

China, Yunnan Province, Diqing Tibetan Autonomous Prefecture.

##### Additional material examined.

China • Yunnan Province, Diqing Tibetan Autonomous Prefecture, Shangri-La City, 27°33'N, 99°49'48"E, altitude 3220 m, isolated from the sclerotium of *O. sinensis*, 16 July 2025, Fang-Wei Lou (living culture KUNCC 11574).

##### Notes.

Phylogenetic analysis based on the combined *tef1-α* and *rpb2* dataset showed that the two samples of *T. diqingense* (HKAS 154753, KUNCC 11574) form a distinct, strongly supported monophyletic lineage within the *Harzianum* clade (ML-BS = 100%, BI-PP = 1.00) (Fig. 1). The species is most closely related to *T. christiani* (CBS 132572), *T. concentricum* (HMAS 248833), *T. ingratum* (HMAS 248822), and *T. perviride* (HMAS 273786). Because no ITS sequences were available for these comparisons, genetic distances were assessed using only *tef1-α* and *rpb2*. *Trichoderma diqingense* differs from *T. christiani* by 1.4% (10/733 bp) in *tef1-α* and 2.5% (27/1,072 bp) in *rpb2*; from *T. concentricum* by 0.3% (2/762 bp) in *tef1-α* and 2.7% (29/1,064 bp) in *rpb2*; from *T. ingratum* by 0.4% (3/762 bp) in *tef1-α* and 1.9% (20/1,064 bp) in *rpb2*; and from *T. perviride* by 0.4% (3/733 bp) in *tef1-α* and 2.5% (27/1,072 bp) in *rpb2*. Morphologically, *T. diqingense* is distinguished by its slender lageniform phialides (6.2–12.4 × 2.2–3.2 μm) compared with those of *T. christiani* (6.0–11.0 × 2.7–3.5 μm), *T. concentricum* (8.6–11.7 × 2.5–3.6 μm), and *T. ingratum* (8.3–12.4 × 2.8–3.5 μm) and by its longer phialides compared with those of *T. perviride* (5.0–8.0 × 1.7–2.3 μm). Its conidia are relatively small and narrowly ellipsoidal (2.4–3.5 × 1.5–2.9 μm), in contrast to the larger, globose to subglobose conidia of *T. christiani* (2.5–3.7 × 2.7–3.0 μm), *T. concentricum* (2.6–3.3 × 2.6–3.3 μm), *T. ingratum* (2.6–3.6 × 2.6–3.2 μm), and *T. perviride* (2.8–3.5 × 2.7–3.3 μm). In addition, chlamydospores are absent in *T. diqingense* but present in *T. christiani* (6.0–9.0 × 5–8 μm) and *T. perviride* (5–6 × 5–5.5 μm). The species also lacks the characteristic coconut-like odor reported for *T. concentricum* and *T. ingratum* (Jaklitsch and Voglmayr 2015; Chen and Zhuang 2017; Qin and Zhuang 2017). Based on the multilocus phylogenetic and detailed morphological evidence, *T. diqingense* is introduced here as a new species.

#### Trichoderma fertile

Taxon classificationFungiHypocrealesHypocreaceae

Bissett, Can. J. Bot. 69(11): 2382 (1991b)

B8B702DA-A286-5A03-9623-8EF7E0EE5134

Fungal Names: FN 359081

Fig. 3

##### Description.

**Sexual morph**: Unknown. **Asexual morph: *Conidiophores*** with a distinct main axis; primary conidiophores relatively stout and elongate, often arising singly from one side of the main axis. Secondary conidiophores short and broad; branches usually opposite or in whorls of three, occasionally solitary. ***Phialides*** ampulliform to subglobose, usually in whorls of 3–6 at the apex of the main axis or each branch, occasionally opposite, rarely solitary, (6.3–)6.3–11.0(–14.2) × (2.2–)2.6–3.6(–4.0) μm (av. 8.4 × 2.9 μm), *l/w* (2.1–)2.2–4.2(–6.1) (av. 2.9). ***Conidia*** smooth-walled, subglobose to globose, light to dark green, (2.4–)2.4–3.4(–4.3) × (1.6–)1.7–2.6(–2.8) μm (av. 2.9 × 2.2 μm), *l/w* (1.0–)1.1–1.6(–2.0) (av. 1.4). ***Chlamydospores*** not observed.

**Figure 3.**
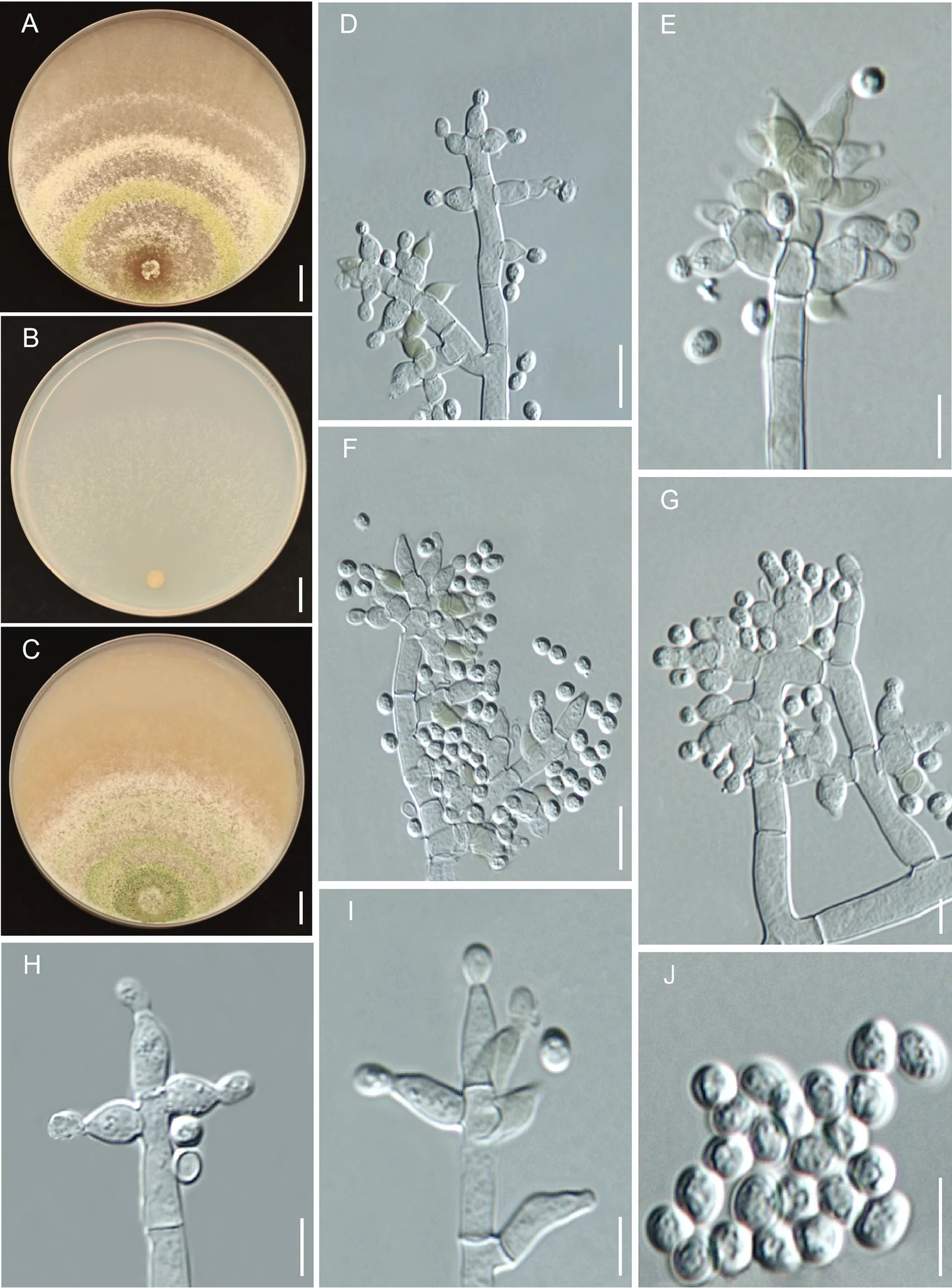
*Trichoderma fertile* (KUNCC 11581, living culture). **A–C**. Cultures on different media after 7 d at 20 °C (A, PDA; B, SNA; C, OA); **D–I**. Conidiophores and phialides; **J**. Conidia. Scale bars: 1 cm **(A–C)**; 10 μm **(D, F, H)**; 5 μm **(E, G, I, J)**.

##### Cultural characteristics.

Colony radius on PDA reached 7.1–7.3 cm after 7 d at 20 °C, covering the plate after 7–8 d. Colony center pale green, margin white; colony margin indistinct; mycelia abundant, compact. Large pustule-like conidial masses with alternating yellow-green and white colors formed after 4 d, becoming dark green after 6 d and forming a semicircular conidial zone around the inoculation point, finally developing into 2–3 broad concentric rings. No diffusing pigment or distinct odor. Colony radius on SNA reached 5.0–5.4 cm after 7 d at 20 °C, covering the plate after 11–12 d. Colonies white; mycelia loose, floccose; colony margin slightly indistinct, fairly regular. Conidiation commenced after 3 d, producing white conidial masses that became yellow-green to dark green after 5 d; concentric rings indistinct. No diffusing pigment or distinct odor. Colony radius on OA reached 7.1–7.4 cm after 7 d at 20 °C, covering the plate after 6–7 d. Center pale green to green, margin white; colony margin regular, slightly fimbriate; mycelia abundant. Large pustule-like conidial masses with alternating white and yellow-green colors formed after 4 d, becoming dark green after 6 d and developing into 3–4 concentric rings around the inoculation point. No diffusing pigment or distinct odor.

##### Distribution.

China, Qinghai Province, Golog Tibetan Autonomous Prefecture (This study), Anhui, Jiangsu, Shandong, and Zhejiang provinces (Jiang et al. 2016); also recorded from New Zealand, Canada, the UK, and the USA (Braithwaite et al. 2017).

##### Material examined.

China • Qinghai Province, Golog Tibetan Autonomous Prefecture, 33°39'N, 99°1'48"E, altitude 4301 m, isolated from the stroma of *O. sinensis*, 12 June 2025, Fang-Wei Lou (dried culture HKAS 155138; living culture KUNCC 11581).

##### Notes.

Phylogenetically, strain KUNCC 11581 was placed in the *Semiorbis* clade, along with the ex-type strain of *T. fertile* (CBS 339.93 and DAOM 167070), with strong statistical support (ML-BS = 100%, BI-PP = 1.00) (Fig. 1). *Trichoderma fertile* was first described from soil under wheat in Canada (Bissett 1991b). Morphological observations and measurements are based on the single culture KUNCC 11581. The strain is morphologically consistent with the protologue of *T. fertile* in conidiophore structure, yellow-green pustulate conidiation, ampulliform phialides, and smooth-walled green conidia. However, it differs from the protologue in having slightly smaller conidia (2.4–3.4 × 1.7–2.6 μm vs. 3.0–4.5 × 1.9–2.5 μm) and in the absence of chlamydospores, which were reported as rare in the protologue (Bissett 1991b). Whether these differences reflect intraspecific variation or culture-induced plasticity remains uncertain because only a single strain was examined. Based on combined morphological and phylogenetic evidence, strain KUNCC 11581 is identified as *T. fertile* and represents a new record from the stroma of *O. sinensis* in China.

#### Trichoderma paraviridescens

Taxon classificationFungiHypocrealesHypocreaceae

Jaklitsch, Samuels & Voglmayr, Persoonia 31: 128 (2013)

F927F495-574B-5966-9EB8-42D00FD09C0A

Fungal Names: FN 803623

Fig. 4

##### Description.

**Sexual morph**: Unknown. **Asexual morph: *Conidiophores*** with a distinct main axis; branches usually arising at right angles to the main axis or directed upward; primary branches mostly opposite or in whorls of three, occasionally solitary. ***Phialides*** ampulliform to conical, rarely curved, usually in whorls of 2–5 at the apex of the main axis and each branch, rarely solitary, (23.3–)23.7–37.1(–39.0) × (4.1–)5.0–7.2(–7.8) μm (av. 29.3 × 6.0 μm), *l/w* (3.6–)3.7–7.0(–7.2) (av. 5.0). ***Conidia*** smooth, obovoid to ellipsoidal, measuring (5.3–)5.7–8.3(–9.5) × (3.8–)3.9–6.6(–6.9) μm (av. 6.8 × 5.2 μm), *l/w* (1.0–)1.0–1.7(–1.8) (av. 1.3). ***Chlamydospores*** terminal or intercalary, globose to subglobose, (5.7–)5.8–20.3(–20.4) × (4.3–)4.7–15.6(–15.6) μm (av. 13.4 × 10.6 μm, *n* = 6), *l/w* (1.1–)1.1–1.4(–1.4) (av. 1.3, *n* = 6).

**Figure 4.**
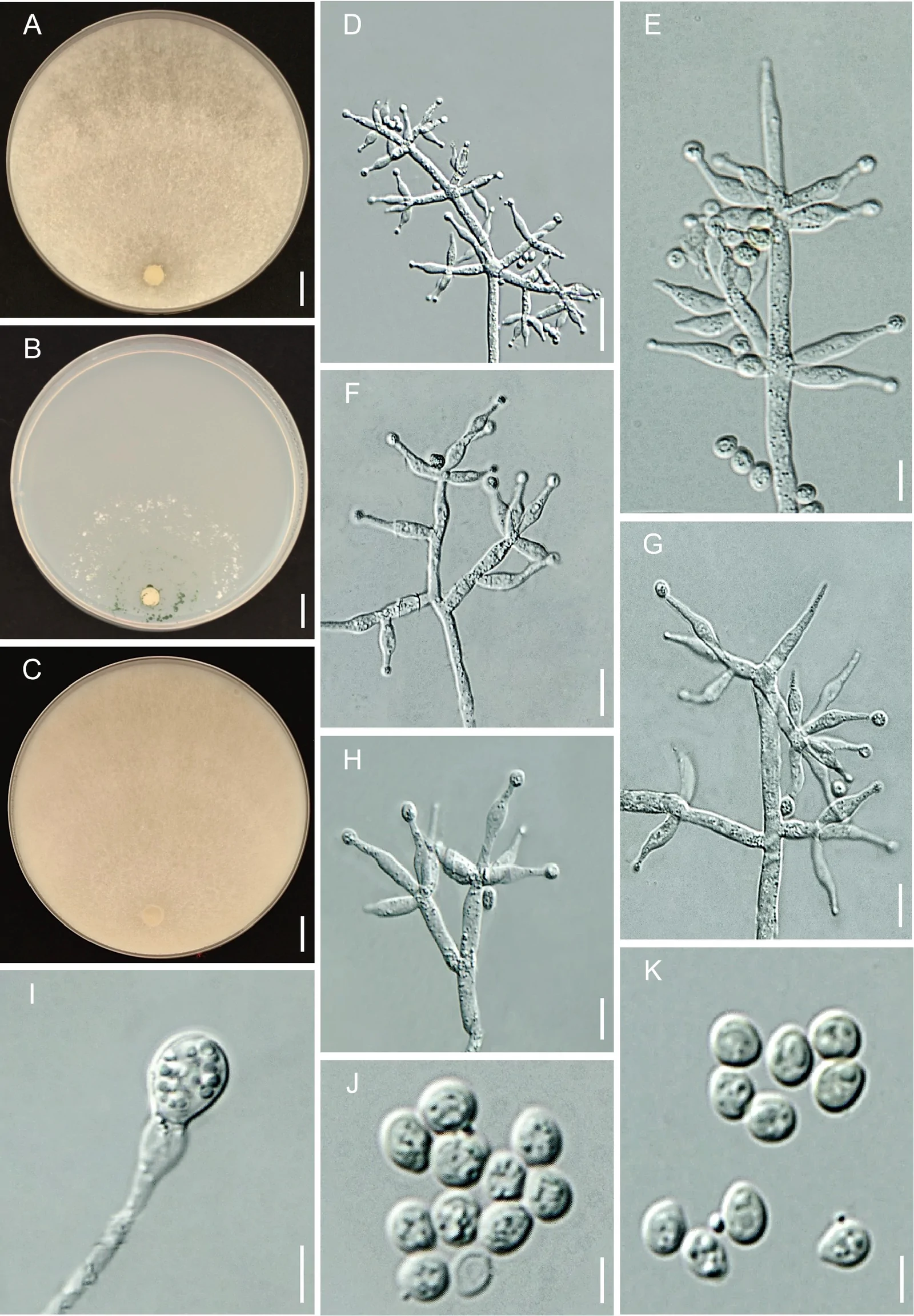
*Trichoderma paraviridescens* (KUNCC 11579, living culture). **A–C**. Cultures on different media after 7 d at 20 °C (A, PDA; B, SNA; C, OA); **D–H**. Conidiophores and phialides; **I**. Chlamydospores; **J, K**. Conidia. Scale bars: 1 cm **(A–C)**; 50 μm **(D)**; 10 μm **(E, I)**; 20 μm **(F–H)**; 5 μm **(J, K)**.

##### Cultural characteristics.

Colony radius on PDA reached 7.1–7.4 cm after 7 d at 20 °C, covering the plate after 7–8 d. Colonies white, radiating; colony margin indistinct; mycelia abundant, dense, composed of narrow hyphae. Conidiation commenced after 9 d as minute yellow-green pustules; conidia became green after 12 d; concentric rings indistinct, with conidiation evenly distributed. No diffusing pigment or distinct odor. Colony radius on SNA reached 3.8–4.1 cm after 7 d at 20 °C, covering the plate after 11–12 d. Colonies well defined, green at the center; colony margin regular, with sparse punctiform white hyphal aggregations; mycelia sparse, floccose, with locally aggregated spots forming a mottled appearance. Conidiation commenced after 4 d as pustules with alternating white and yellow-green colors; conidia became green after 6 d, and the pustules eventually formed 2–3 indistinct concentric rings. No diffusing pigment or distinct odor. Colony radius on OA reached 7.0–7.3 cm after 7 d at 20 °C, covering the plate after 7–8 d. Colonies white, radiating, with indistinct margins; mycelia sparse. Conidiation commenced after 14 d as pustules with alternating white and yellow-green colors, evenly distributed and lacking distinct concentric rings. No diffusing pigment or distinct odor.

##### Distribution.

China, Xizang Autonomous Region (this study), Gansu Province (Zeng et al. 2020); widely distributed in Europe; also known from Colombia, Iran, Japan, Mexico, and the USA (Jaklitsch et al. 2013).

##### Material examined.

China • Xizang Autonomous Region, Nyingchi City, 28°48'N, 95°54'36"E, altitude 2300 m, isolated from the stroma of *O. sinensis*, 21 July 2025, Fang-Wei Lou (dried culture HKAS 155137; living culture KUNCC 11579).

##### Notes.

Phylogenetically, strain KUNCC 11579 was placed in the *Viride* clade, forming a subclade with the ex-type strains of *T. inconspicuum* (YMF 1.04623) and *T. paraviridescens* (CBS 119321) (ML-BS = 100%, BI-PP = 0.52) (Fig. 1). Strain KUNCC 11579 shares nearly identical sequences with both *T. inconspicuum* and *T. paraviridescens*. It differs from *T. inconspicuum* by 0.60% (6/979 bp) in *tef1-α* and 0.57% (6/1,048 bp) in *rpb2*, and from *T. paraviridescens* by 0.60% (6/1,002 bp, including 3 gaps) in *tef1-α* and 0.55% (6/1,093 bp) in *rpb2*. ITS differs from *T. inconspicuum* by 0.17% (1/597 bp) and from *T. paraviridescens* by 0.35% (2/572 bp, including 2 gaps). However, ITS alone offers limited resolution for species-level discrimination in *Trichoderma*, especially among closely related taxa (Cai and Druzhinina 2021). Morphologically, strain KUNCC 11579 differs from *T. inconspicuum* in having longer phialides (23.7–37.1 × 5.0–7.2 μm vs. 6.4–14.4 × 1.9–4.0 μm) and larger conidia (5.7–8.3 × 3.9–6.6 μm vs. 2.8–4.5 × 2.6–4.0 μm) (Zheng et al. 2021). In contrast, strain KUNCC 11579 is broadly consistent with the protologue of *T. paraviridescens* in conidiophores, phialides, chlamydospores, and conidia. *Trichoderma paraviridescens* was originally described from the decorticated wood of *Fagus sylvatica* in Austria and is known from wood-, bark-, and fungus-associated substrates (Jaklitsch et al. 2013). Therefore, although strain KUNCC 11579 is phylogenetically close to *T. inconspicuum*, its morphological divergence from that species, together with its general agreement with *T. paraviridescens*, supports its identification as the latter and represents a new substrate record from the stroma of *O. sinensis* in China.

#### Trichoderma dracaenae

Taxon classificationFungiHypocrealesHypocreaceae

F. W. Lou, Zhu L. Yang & Y. B. Wang sp. nov.

ABA3BA36-83E6-50D2-A931-A7B274B269BC

Fungal Names: FN 573938

Fig. 5

##### Etymology.

Named after the plant genus *Dracaena*, from which this species was isolated.

**Figure 5.**
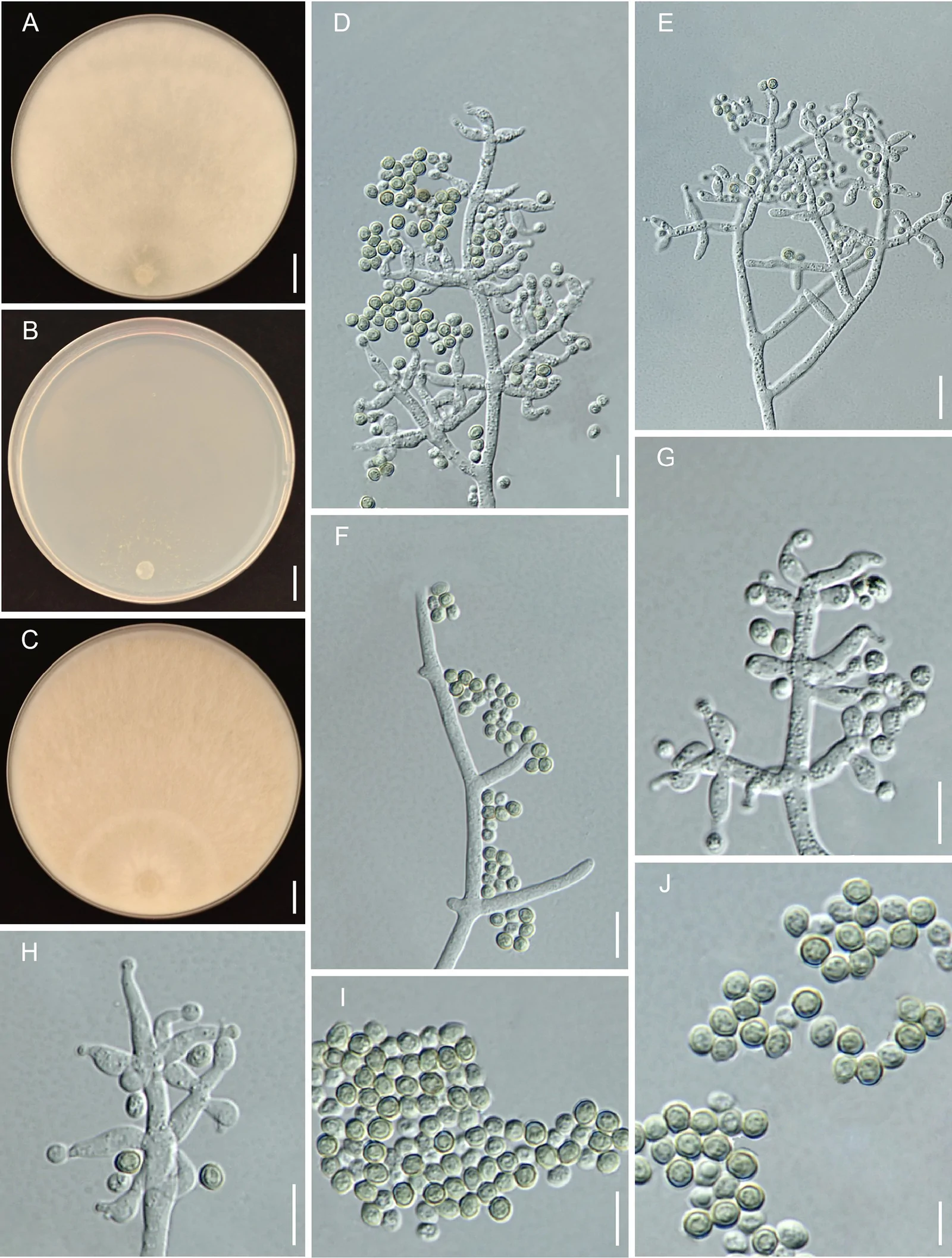
*Trichoderma dracaenae* (KUNCC 11575, ex-type living culture). **A–C**. Cultures on different media after 7 d at 20 °C (A, PDA; B, SNA; C, OA); **D–H**. Conidiophores and phialides; **I, J**. Conidia. Scale bars: 1 cm **(A–C)**; 10 μm **(D–H)**; 5 μm **(I, J)**.

##### Type.

China • Guangxi Zhuang Autonomous Region, Baise City, 24°36'N, 106°16'12"E, altitude 688 m, isolated from the healthy root of *Dracaena* sp., 4 March 2025, Fang-Wei Lou (holotype HKAS 154754; ex-type living culture KUNCC 11575).

##### Diagnosis.

*Trichoderma dracaenae* is similar to *T. sphaerosporum* and *T. speciosum* but differs in having conidiophores with a distinct cylindrical main axis and usually 2–3 branches, broader ampulliform phialides, and smaller, smooth-walled, subglobose to globose conidia.

##### Description.

**Sexual morph**: Unknown. **Asexual morph: *Conidiophores*** cylindrical, with an obvious main axis, usually bearing 2–3 branches in whorls on the main axis or arising singly. ***Phialides*** usually arranged in whorls of 3–5 at the apex of the main axis or each branch, rarely solitary, ampulliform, usually curved toward the apex, (4.9–)5.3–11.6(–13.6) × (1.9–)2.1–3.6(–3.8) μm (av. 8.8 × 2.8 μm), *l/w* (1.5–)1.7–4.9(–5.6) (av. 3.3). ***Conidia*** smooth-walled, subglobose to globose, green, (2.6–)2.8–3.4(–3.7) × (2.4–)2.6–3.1(–3.2) μm (av. 3.1 × 2.8 μm), *l/w* (1.0–)1.1–1.2(–1.3) (av. 1.1). ***Chlamydospores*** not observed.

##### Cultural characteristics.

Colony radius on PDA reached 7.5–7.8 cm after 7 d at 20 °C, covering the plate after 6–7 d. Colonies white, radiating, with indistinct margins; mycelia abundant, dense; concentric rings absent. Conidiation absent. No diffusing pigment or distinct odor. Colony radius on SNA reached 2.7–3.0 cm after 7 d at 20 °C, covering the plate after 16–17 d. Colonies hyaline, with sparse, pale-white mycelia; colony margin indistinct. Conidiation commenced after 8 d as minute white pustules; conidia gradually became green after 10 d, with concentric rings indistinct. No diffusing pigment or distinct odor. Colony radius on OA reached 7.1–7.4 cm after 7 d at 20 °C, covering the plate after 7–8 d. Colonies white, radiating, with indistinct margins; mycelia abundant, floccose; concentric rings indistinct. Conidiation commenced after 18 d as minute white pustules; conidia gradually became green after 22 d. No diffusing pigment or distinct odor.

##### Distribution.

China, Guangxi Zhuang Autonomous Region, Baise City.

##### Additional material examined.

China • Guangxi Zhuang Autonomous Region, Baise City, 24°36'N, 106°16'12"E, altitude 688 m, isolated from the healthy root of *Dracaena* sp., 4 March 2025, Fang-Wei Lou (living culture KUNCC 11576).

##### Notes.

Phylogenetic analysis placed the two samples of *T. dracaenae* (HKAS 154754, KUNCC 11576) in a well-supported monophyletic lineage within the *Viride* clade, sister to *T. speciosum* (YMF 1.00205) and *T. sphaerosporum* (HMAS 273763) (ML-BS = 100%, BI-PP = 1.00) (Fig. 1). Because no ITS sequences were available for comparison, genetic distances were assessed using only *tef1-α* and *rpb2*. *Trichoderma dracaenae* can be distinguished from *T. speciosum* by 1.8% (17/925 bp, including 1 gap) in *tef1-α* and 2.3% (20/868 bp) in *rpb2* and from *T. sphaerosporum* by 0.79% (8/1,018 bp, including 1 gap) in *tef1-α* and 2.4% (17/906 bp) in *rpb2*. Morphologically, *T. dracaenae* has conidiophores with a distinct cylindrical main axis and usually 2–3 branches, differing from the tree-type conidiophores with paired or whorled branches in *T. speciosum*, and the typical *Trichoderma*-type conidiophores of *T. sphaerosporum*, which sporulates rapidly on CMD and PDA within 2–3 days. Phialides of *T. dracaenae* are ampulliform, usually curved toward the apex, and broader (5.3–11.6 × 2.1–3.6 μm) than those of *T. speciosum* (5.0–10.0 × 2.0–3.0 μm) and *T. sphaerosporum* (6.5–10 × 2–2.5 μm). Its conidia are smooth-walled, subglobose to globose, and smaller (2.8–3.4 × 2.6–3.1 μm) than those of *T. speciosum* (3.7–4.9 × 3.1–3.8 μm), which are verrucose. In addition, *T. dracaenae* lacks the distinct coconut-like odor reported for *T. sphaerosporum* (Qin and Zhuang 2016; Qiao et al. 2018). Based on multilocus phylogenetic and morphological evidence, *T. dracaenae* is described here as a new species.

#### Trichoderma yuxiense

Taxon classificationFungiHypocrealesHypocreaceae

F. W. Lou, Zhu L. Yang & Y. B. Wang sp. nov.

32C66AFE-E9C6-5076-9479-731311CE8C8B

Fungal Names: FN 573936

Fig. 6

##### Etymology.

Named after the location, Yuxi, where this species was collected.

**Figure 6.**
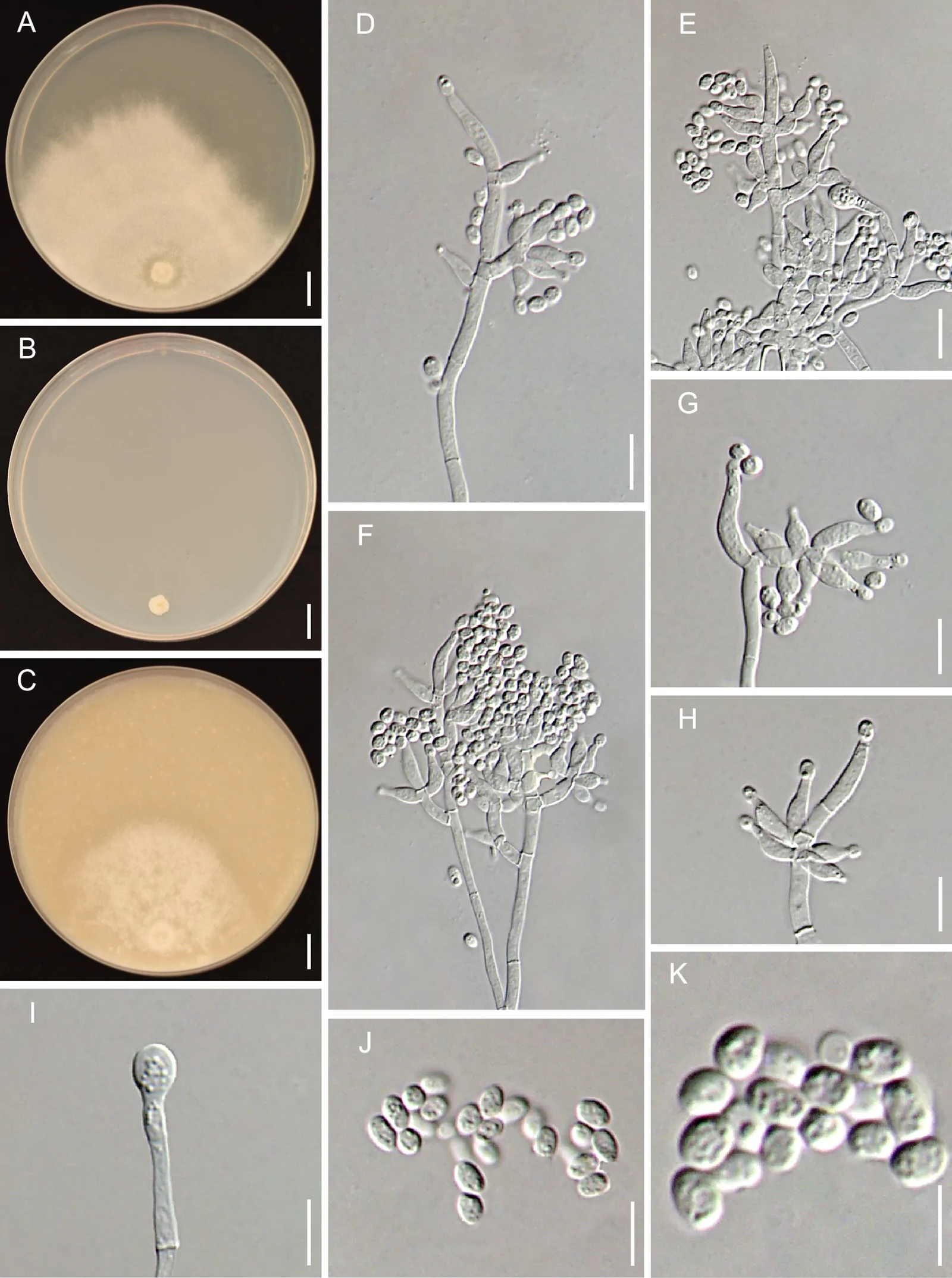
*Trichoderma yuxiense* (KUNCC 11571, ex-type living culture). **A–C**. Cultures on different media after 7 d at 20 °C (A, PDA; B, SNA; C, OA); **D–H**. Conidiophores and phialides; **I**. Chlamydospores; **J, K**. Conidia. Scale bars: 1 cm **(A–C)**; 10 μm **(D–H)**; 5 μm **(I–K)**.

##### Type.

China • Yunnan Province, Yuxi City, 23°33'36"N, 101°18'E, altitude 2434 m, isolated from the sclerotium of *Ophiocordyceps jinguangensis*, 26 July 2024, Fang-Wei Lou (holotype HKAS 154752; ex-type living culture KUNCC 11571).

##### Diagnosis.

This species is similar to *T. parapiluliferum* and *T. shangrilaense* but differs in having longer and narrower ampulliform to lageniform phialides, subglobose to broadly ellipsoidal conidia, and rare, mostly terminal chlamydospores, which are absent in *T. shangrilaense*.

##### Description.

**Sexual morph**: Unknown. **Asexual morph: *Conidiophores*** with an obvious main axis, usually asymmetrically branched, occasionally unbranched; branches diverging from the main axis at angles slightly less than 90°. ***Phialides*** rarely solitary, mostly in whorls of 2–6 on the main axis and branches, ampulliform to lageniform, (7.5–)8.0–11.1(–12.1) × (1.7–)1.9–2.9(–3.2) μm (av. 9.6 × 2.5 μm), *l/w* (2.6–)2.8–5.3(–7.0) (av. 4.0). ***Conidia*** smooth-walled, hyaline, subglobose to broadly ellipsoidal, (2.0–)2.4–3.5(–3.7) × (1.2–)1.5–2.5(–2.9) μm (av. 2.9 × 2.1 μm), *l/w* (1.0–)1.1–1.9(–2.5) (av. 1.5). ***Chlamydospores*** rare, mostly terminal, globose to subglobose, (2.8–)3.6–7.2(–7.6) × (2.0–)2.4–5.3(–5.4) μm (av. 5.4 × 4.0 μm, *n* = 9), *l/w* (1.1–)1.2–1.9(–1.9) (av. 1.4, *n* = 9).

##### Cultural characteristics.

Colony radius on PDA reached 5.3–5.6 cm after 7 d at 20 °C, covering the plate after 11–12 d. Colonies well defined, cream-white; colony margin irregular, radiate, distinct; mycelia abundant, dense. Large white pustules formed after 11 d. Concentric rings indistinct. No diffusing pigment or distinct odor. Colony radius on SNA reached 1.6–1.9 cm after 7 d at 20 °C, covering the plate after 25–27 d. Colonies white, flat, thin; colony margin indistinct, without obvious features; mycelia sparse. Conidiation not observed. No diffusing pigment or distinct odor. Colony radius on OA reached 3.3–3.6 cm after 7 d at 20 °C, covering the plate after 13–14 d. Colonies white, colony margin regular, slightly radiate with a fimbriate edge; mycelia abundant, floccose, composed of loose hyphae. Small white pustules formed after 10 d. Conidiation sparse; concentric rings indistinct. No diffusing pigment or distinct odor.

##### Distribution.

China, Yunnan Province, Yuxi City.

##### Additional material examined.

China • Yunnan Province, Yuxi City, 23°33'36"N, 101°18'E, altitude 2434 m, isolated from the sclerotium of *O. jinguangensis*, 26 July 2024, Fang-Wei Lou (living culture KUNCC 11572).

##### Notes.

Phylogenetic analysis of the combined ITS, *tef1-α*, and *rpb2* dataset placed the two isolates of *T. yuxiense* (HKAS 154752, KUNCC 11572) in a well-supported lineage within the *Polysporum* clade (ML-BS = 100%, BI-PP = 1.00) (Fig. 1). The species is most closely related to *T. parapiluliferum* (GJS 91-60) and *T. shangrilaense* (WT34004). However, *T. yuxiense* differs from *T. parapiluliferum* by 2.2% (17/329 bp, including 4 gaps) in *tef1-α* and 2.8% (30/1,087 bp, including 1 gap) in *rpb2* and from *T. shangrilaense* by 2.5% (25/1,004 bp, including 2 gaps) in *tef1-α* and 2.7% (29/1,099 bp, including 2 gaps) in *rpb2*. The ITS sequences are identical to those of *T. parapiluliferum* (0/580 bp) and *T. shangrilaense* (0/574 bp). Morphologically, *T. yuxiense* differs from *T. parapiluliferum* and *T. shangrilaense* in three main features. Its phialides are longer and narrower (8.0–11.1 × 1.9–2.9 μm) than those of *T. parapiluliferum* (4.5–7.5 × 2.4–3.5 μm) and *T. shangrilaense* (5.7–9.0 × 3.2–3.5 μm), the latter being flask-shaped and often curved. Its conidia are subglobose to broadly ellipsoidal (2.4–3.5 × 1.5–2.5 μm), whereas *T. parapiluliferum* has ellipsoidal to oblong conidia and *T. shangrilaense* has obovoid to ellipsoidal conidia (3.5–4.0 × 3.0–3.3 μm). Chlamydospores are rare and mostly terminal in *T. yuxiense* (3.6–7.2 × 2.4–5.3 μm), similar to those of *T. parapiluliferum* (6.0–8.5 × 5.5–8.0 μm), but absent in *T. shangrilaense* (Lu et al. 2004; Jaklitsch and Voglmayr 2013; Zhang et al. 2022). Therefore, based on the concordant evidence from phylogeny and morphology, *T. yuxiense* is proposed here as a new species.

## Discussion

China is a global hotspot for fungal diversity, with 1,249 new species described in 2023 alone, representing 41.98% of the global total (Wang et al. 2024). However, only approximately 6.84% of the known global fungal diversity has been recorded from China, suggesting that numerous species remain to be discovered (Wang and Cai 2023). *Trichoderma* is one of the most studied fungal genera globally and has also been widely examined in China (Cai and Druzhinina 2021). A soil survey in China reported 23 new species (Chen and Zhuang 2017), and subsequent taxonomic studies have reported additional *Trichoderma* taxa from various regions and diverse substrates across the country (Zhao et al. 2023, 2025; Wang et al. 2025).

Herein, eight *Trichoderma* strains from *Ophiocordyceps* spp. and *Dracaena* sp. were delimited based on phylogenetic and morphological evidence. However, the taxonomy and species identification of *Trichoderma* still face considerable challenges. Morphological characters alone are often insufficient to distinguish closely related species; accurate identification depends on three DNA barcodes (ITS, *tef1-α*, and *rpb2*), but reference sequences remain incomplete for many taxa. Moreover, species delimitation frequently involves subjective judgment, leading to considerable ambiguity in the molecular identification of a substantial proportion of *Trichoderma* species (Cai and Druzhinina 2021). Given these challenges, species delimitation in this study was conducted using ITS, *tef1-α*, and *rpb2* sequence data, together with morphological evidence. The results showed that the eight isolates obtained in this study were assigned to four clades of *Trichoderma*, namely the *Harzianum*, *Semiorbis*, *Viride*, and *Polysporum* clades. The results identified three new species, namely *T. diqingense*, *T. dracaenae*, and *T. yuxiense* and two new substrate records, *T. fertile* and *T. paraviridescens*.

In the *Harzianum* clade, *T. diqingense* has regularly pyramidal conidiophores, phialides of varying dimensions, and smaller ellipsoidal conidia, which distinguish it from *T. christiani*, *T. concentricum*, *T. ingratum*, and *T. perviride*; it also lacks chlamydospores, which are present in *T. christiani* and *T. perviride*, and does not produce the coconut-like odor reported for *T. ingratum* (Jaklitsch and Voglmayr 2015; Chen and Zhuang 2017; Qin and Zhuang 2017). *Trichoderma dracaenae*, belonging to the *Viride* clade, is distinguished from *T. sphaerosporum* and *T. speciosum* by conidiophores with a distinct main axis and typically 2–3 branches, broader phialides, and smaller, smooth-walled conidia (Qin and Zhuang 2016; Qiao et al. 2018). *Trichoderma yuxiense*, within the *Polysporum* clade, differs from *T. parapiluliferum* and *T. shangrilaense* in having longer and narrower ampulliform to lageniform phialides, smaller subglobose to broadly ellipsoidal conidia, and rare, mostly terminal chlamydospores (Lu et al. 2004; Jaklitsch and Voglmayr 2013; Zhang et al. 2022). Morphological and molecular evidence supports the delimitation of the three new species.

Of the eight isolates, six were obtained from *Ophiocordyceps* tissues and two from *Dracaena* roots. *Trichoderma fertile* and *T. paraviridescens* are newly recorded from the stromata of *O. sinensis*, thereby extending their known substrate ranges (Chaverri and Samuels 2003; Jaklitsch 2009; Chen and Zhuang 2017). *Trichoderma* species have previously been isolated from the stromata of *O. sinensis* (Zhang et al. 2020), which is slow-growing *in vitro* and consequently prone to overgrowth by faster-growing saprobes (Baral and Maharjan 2012). Although *Trichoderma* species are fast-growing and theoretically capable of influencing community composition through resource competition (Bissett 1991a; Guzmán-Guzmán et al. 2017; Dou et al. 2022), no experimental data on ecological interactions were obtained in the present study, and their precise roles in *Ophiocordyceps*-associated systems thus remain unresolved.

In summary, this study expands the documented species diversity of *Trichoderma*, reports new substrate occurrences from *Ophiocordyceps* materials, and provides a foundation for understanding the distribution of the genus across diverse ecological substrates. Nonetheless, this study is constrained by the limited number of isolates, the potential for morphological plasticity under culture conditions, and the inherent difficulty of inferring *in situ* ecological interactions from culture-based data alone. Future work should broaden geographic and substrate sampling to obtain more representative isolates and integrate multilocus phylogenetic analyses (e.g., incorporating additional nuclear gene loci) and phylogenomic approaches to more accurately delimit species boundaries within *Trichoderma* and clarify its distribution patterns and ecological associations across diverse substrates.

## Supplementary Material

XML Treatment for Trichoderma diqingense

XML Treatment for Trichoderma fertile

XML Treatment for Trichoderma paraviridescens

XML Treatment for Trichoderma dracaenae

XML Treatment for Trichoderma yuxiense

## Additional information

### Artificial Intelligence (AI) use

The authors accept full responsibility for the content of the manuscript, including the disclosure of any use of AI.

No AI tools were used in the preparation of this manuscript.

### Author ORCIDs

F.-W. Lou https://orcid.org/0009-0003-9065-7443

B.-Z. Chen https://orcid.org/0009-0005-7214-4913

L.-M. Tan https://orcid.org/0009-0006-9600-156X

J.-C. Xiao https://orcid.org/0009-0004-6686-2110

H.-F. Liao https://orcid.org/0009-0006-7287-1384

J.-H. Hou https://orcid.org/0009-0008-8691-1011

Z.-L. Yang https://orcid.org/0000-0001-9745-8453

G.-D. Li https://orcid.org/0000-0002-9108-5454

Y.-B. Wang https://orcid.org/0000-0002-3305-9418

### Data availability

All data supporting the findings of this study are included in the Supplementary materials.

## Supplementary materials

Supplementary material 1 Supplementary tables Data type xlsx Explanation note **table S1**. Specimen information and GenBank accession numbers for sequences used in Suppl. material 2. **table S2**. Specimen information and GenBank accession numbers for sequences used in Fig. 1. **table S3**. Best-fitting models selected for each gene partition for use in Maximum Likelihood (ML) and Bayesian Inference (BI) analyses across species of *Trichoderma*. **table S4**. Morphological comparison of *Trichoderma* species closely related to those in this study based on phylogenetic analysis. https://binary.pensoft.net/file/1751601This dataset is made available under the Open Database License (http://opendatacommons.org/licenses/odbl/1.0/). The Open Database License (ODbL) is a license agreement intended to allow users to freely share, modify, and use this Dataset while maintaining this same freedom for others, provided that the original source and author(s) are credited. Fang-Wei Lou, Bao-Zheng Chen, Li-Man Tan, Jia-Chen Xiao, He-Fa Liao, Jian-Hua Hou, Zhu-Liang Yang, Guo-Dong Li, Yuan-Bing Wang

Supplementary material 2 Phylogenetic tree Data type pdf https://binary.pensoft.net/file/1751602This dataset is made available under the Open Database License (http://opendatacommons.org/licenses/odbl/1.0/). The Open Database License (ODbL) is a license agreement intended to allow users to freely share, modify, and use this Dataset while maintaining this same freedom for others, provided that the original source and author(s) are credited. Fang-Wei Lou, Bao-Zheng Chen, Li-Man Tan, Jia-Chen Xiao, He-Fa Liao, Jian-Hua Hou, Zhu-Liang Yang, Guo-Dong Li, Yuan-Bing Wang
