# Supplementary material for: Molecular phylogeny and morphology reveal three new species and two new records of Trichoderma (Hypocreaceae, Hypocreales) from China
Source: MycoKeys. 2026 Aug 21;139:23–47. doi: 10.3897/mycokeys.139.198944 (PMC13525404; doi:10.3897/mycokeys.139.198944)
Supplement: Supplementary file 1 [file mycokeys-139-023-s002.pdf]

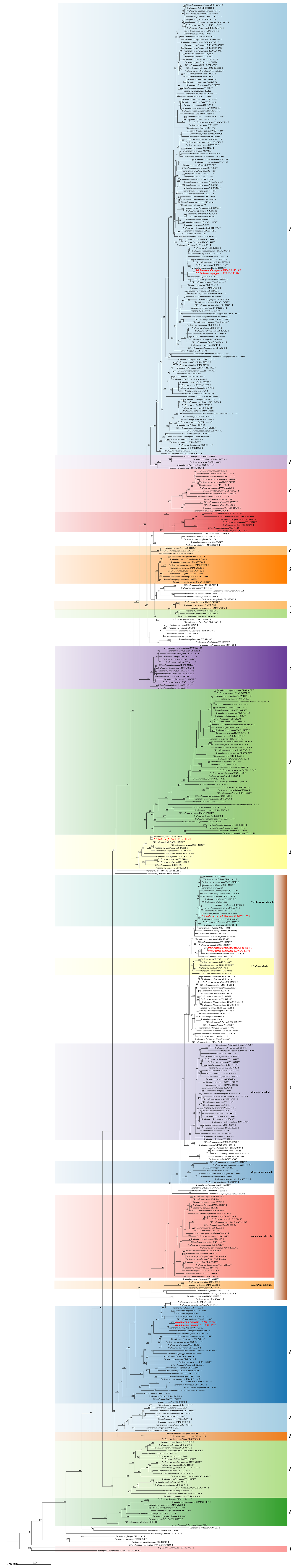
Harzianum
Helicum
Chlorosporum
Spinulosum
Ceramicum
Strictipile
Hunanense
Spirale
Stromaticum
Longibrachiatum
Semiorbis
Viride
Polysporum
Brevicompactum
Deliquescens
Hypocreanum
Psychrophilum
Outgroup
Tree scale
0.04
